# A Substage Increase in The AJCC Classification System Improves Prognostic Prediction in Stage III Gastric Cancer With Insufficient Lymph Nodes Removed

**DOI:** 10.3389/fonc.2021.624413

**Published:** 2021-03-05

**Authors:** Ri-Sheng Zhao, Yi-Nan Liu, Wei-Gang Dai, Si-Le Chen, Jin-Ning Ye, Er-Tao Zhai, Shi-Rong Cai, Jian-Hui Chen

**Affiliations:** ^1^Division of Gastrointestinal Surgery Center, The First Affiliated Hospital, Sun Yat-sen University, Guangzhou, China; ^2^Gastric Cancer Center, Sun Yat-sen University, Guangzhou, China

**Keywords:** gastric cancer, stage III, lymph nodes removed, overall survival, stage migration

## Abstract

**Background:**

The impact of lymph nodes (LNs) removed on the survivals of patients with stage III gastric cancer, especially on that of those who undergo the adjuvant chemotherapy as a compensation for a possibly insufficient lymphadenectomy, is still unclear.

**Methods:**

Consecutive patients (n = 488) with stage III gastric cancer under R0 curative resection followed by adjuvant chemotherapy were analyzed. The overall survival (OS) was compared between patients with insufficient LNs removed (ILNr, <16 LNs) and sufficient LNs removed (SLNr, ≥16 LNs). Performance of the prediction systems was evaluated using the Likelihood ratio χ^2^ test, Akaike information criterion (AIC), Harrell’s concordance index (C-index), and area under the receiver operating characteristic curves (AUC).

**Results:**

The OS of patients were significantly longer in those with SLNr relative to those with ILNr (for stage IIIA, 68.2 vs. 43.2 months, *P* = 0.042; for stage IIIB, 43.7 vs. 24.9 months, *P* < 0.001; for stage IIIC, 23.9 vs. 8.3 months, *P* < 0.001; and for total stage III, 37.7 vs. 21.7 months, *P* < 0.001). However, the OS were similar between stage IIIA patients with ILNr and stage IIIB patients with SLNr (*P* = 0.928), between IIIB patients with ILNr and IIIC patients with SLNr (*P* = 0.962), and IIIC patients with ILNr and stage IV (*P* = 0.668), respectively. A substage increase in the AJCC classification system, from IIIA to IIIB, from IIIB to IIIC, and from IIIC to IV in patients with ILNr, enhanced the accuracy of prognostic prediction in patients with stage III gastric cancer compared to the current TNM system (Likelihood ratio χ^2^, 188.6 vs. 184.8; AIC, 4336.4 vs. 4340.6; C-index, 0.695 vs. 0.679, *P* = 0.002). The ROC curves revealed that the performance of prognostic prediction was better in the new prediction system (AUC = 0.699) compared with the current TNM system (AUC = 0.676).

**Conclusions:**

ILNr (LNs <16) impairs the long-term outcomes of stage III gastric cancer underwent adjuvant chemotherapy. The status of LNs removal adds values to the current TNM system in prognostic prediction of stage III gastric cancer.

## Introduction

Gastric cancer is the fifth most common cancer and the third leading cause of cancer-related death globally. Almost 50% of reported cases are from eastern Asia and a majority are from China (1). Although the efficacy of multiple therapies has led to improvement in the treatment of gastric cancer over the years (2), R0 resection with standard lymphadenectomy that regularly involves removal of the regional lymph nodes (LNs) surrounding the stomach is still the most effective treatment for advanced diseases (3). Metastatic LNs are found in 74.4% of patients with advanced gastric cancer (4). The number and extent of metastatic LNs has been demonstrated to negatively correlate with the long-term survival of patients (5). Increasing the number of LNs removed during lymphadenectomy is theoretically an effective way to ensure a higher probability of completely removing the tumour-draining LNs. Some studies have revealed that a higher retrieved number of LNs was associated with better survival outcomes in patients with gastric cancer (6, 7). Notably, there are evidence that suggest that removing more LNs also provides protection to patients without LN metastasis (8). Some evidence even showed that the benefits of protection was more obvious in patients with less advanced gastric cancer compared to those with more advanced disease (9). Taken together, patients with gastric cancer would benefit from the removal of sufficient LNs.

The TNM staging system is currently the most useful tool for predicting the prognosis of gastric cancer (10). In the TNM system, the T and M staging can be accurately scored based on the pathological and radiological examinations, but challenges still exist in the N staging because of the inconsistence in accuracy induced by variations in the number of LNs removed. Although the N-ratio (the ratio of metastatic LNs in the total LNs examined) was employed to compensate for the insufficient removal of LNs and was demonstrated to be valuable in the prognostic prediction of gastric cancer (11, 12), this ratio is not comparable to increasing the number of LNs removed to allow more accurate assessment of LNs involvement. Since the number of LNs removed is critical to both improving and prediction of the prognosis, we assessed whether the status of LNs removed may have a direct impact on the overall survival (OS) in patients with stage III gastric cancer that predominantly have LN metastasis, especially in those who underwent adjuvant chemotherapy as a compensation for a possibly insufficient lymphadenectomy.

The eighth edition of the American Joint Committee on Cancer (AJCC) staging system recommends N staging as follows: N0, 0 positive LN; N1, 1–2 positive LNs; N2, 3–6 positive LNs; N3a, 7–15 LNs; N3b, ≥ 16 LNs (13). According to this guideline, at least 16 LNs assessed per patient are sufficient for N stage assignment. Thus, we classified the status of LNs removed into two categories: sufficient LNs removed (SLNr, when the number of LNs removed was ≥ 16) and insufficient LNs removed (ILNr, when the number was < 16). In this study, we reviewed 488 consecutive patients undergoing D2 lymphadenectomy with R0 curative resection and were pathologically diagnosed as stage III and received adjuvant chemotherapy. Stratified analyses were performed on the association between the status of LNs removed and OS in patients with IIIA, IIIB, and IIIC diseases. This study will allow us to have a view on the value of the status of LNs removed beyond the TNM system in prognostic prediction of patients with stage III gastric cancer.

## Methods

### Patient Population

**Figure 1:**
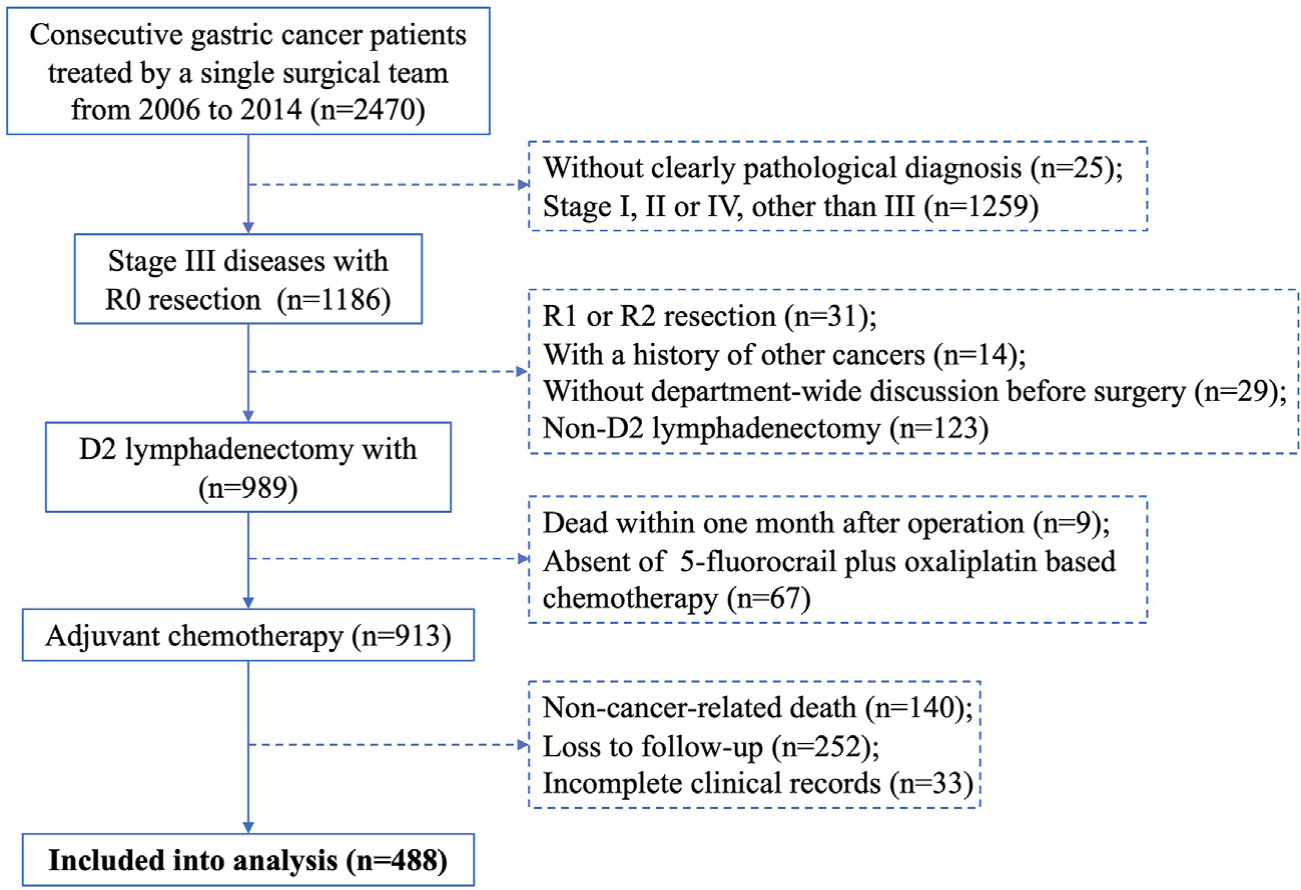
Flowchart of the case screening process.

Consecutive patients with primary gastric cancer treated by a single surgical team at The First Affiliated Hospital of Sun Yat-sen University between January 2006 and December 2014 were individually reviewed by two researchers. Cases included meet the following criteria: (1) D2 lymphadenectomy with R0 resection, (2) pathological diagnosis of stage III gastric cancer, (3) underwent 5-fluorocrail plus oxaliplatin-based adjuvant chemotherapy, (4) discussed in a department-wide conference before operation, (5) complete clinical records, including age, gender, neoadjuvant chemotherapy, tumour features (site, size, Borrmann type, status of differentiation), pathological T, N and M staging, surgeon who performed the operation, CEA level and surgical complications (surgical site infection, anastomotic stricture, anastomotic leakage, anastomotic bleeding, abdominal bleeding, pleural effusion), and (6) complete follow-up records. Cases were excluded if they had a history of other cancers, non-cancer-related death, or died within one month after surgery. Discrepancies in inclusion were resolved by a principal investigator. A flowchart depicting the case screening process of this study is shown in **Figure 1**.

TNM staging was determined according to the eighth edition of the AJCC staging system. The definition of D2 lymphadenectomy was consistent with the Japanese Gastric Cancer Association guidelines (3). Eight continuous cycles of XELOX or SOX regimen were used in the adjuvant chemotherapy that was commenced within 1 month postoperatively. For each cycle of XELOX, the patients were administered intravenous oxaliplatin (130 mg/m^2^) on day 1 and orally administered capecitabine (1000 mg/m^2^, twice daily) on days 1–14, followed by a rest period until day 21. For each cycle of SOX, the regimen was the same as XELOX except for capecitabine that was substituted by S-1 (80 mg, 100 mg, or 120 mg corresponding to the body-surface area <1.25 m^2^, ≥1.25 m^2^ but <1.5 m^2^, and ≥1.5 m^2^, respectively). Senior surgeons were deemed to have 10 or more years of experience performing gastrectomies. Follow-up by outpatient or telephone interview was undertaken every three months in the first two years after surgery, every 6 months from the third year to the fifth year, and once per year thereafter. OS was calculated based on the follow-up results. The follow-up information of this study was collected until December 2019. This study adhered to the guidelines approved by the Hospital Ethics Committee.

### Data Analysis

The chi-square test and Mantel-Haenszel chi-square test were used to compare unordered categorical variables between patients with SLNr and those with ILNr, while Spearman’s correlation coefficient was used to compare the ordered categorical variables. The Cox proportional hazards model was employed in multivariate analysis of the independent factors that influence the OS of patients. Univariate survival analysis was performed to compare the OS between different substages of patients using the Kaplan-Meier method and Log-rank test. Multivariate logistic regression was used to assess the risk factors for ILNr (α = 0.1 was adopted for stepwise regression). To evaluate the prediction system that integrates TNM staging with the status of LNs removed, the Likelihood ratio χ^2^ test was employed to measure the homogeneity of survival times among the same subset of cases. We further calculated the Akaike information criterion (AIC), which compared the relative amount of information lost by the prognostic systems (14). The less the AIC, the less the information lost and the higher the quality of the model was (14). All the above statistical analyses were performed using the Statistical Package for Social Science (SPSS, version 25.0, Chicago, USA). Moreover, the Harrell’s concordance index (C-index) was calculated using the ‘survcomp’ packages in R 4.0.0 software to assess the discriminatory ability of the model (15). There was a positive correlation between the c-index and the proportion of correct predictions (15). The receiver operating characteristic (ROC) curve and the area under the curve (AUC) were used to compare the performance of the proposed prediction system with the conventional TNM system. *P*-*values* less than 0.05 indicated that differences were statistically significant.

## Results

### Clinicopathological Characteristics

Out of the 488 patients with stage III tumours, 128 (26.2%) had stage IIIA, 189 (38.7%) had stage IIIB, and 171 (35.0%) had stage IIIC. For the status of LNs removed, 89 (18.2%) patients were has ILNr and 389 (81.8%) had with SLNr. The median age of the included patients was 57.8 years (range, 24–87 years). The clinicopathological characteristics of the included patients with ILNr or SLNr are shown in **Table 1**. Patients with SLNr were significantly associated with smaller tumour size, more advanced N and TNM stage, and more operations performed by senior surgeons (all *P* < 0.05). There was no significant difference between patients with SLNr and those with ILNr in neoadjuvant chemotherapy, tumour sites, gross type of tumours, degree of tumour differentiation, T stage of tumours and CEA levels (all *P* > 0.05).

**Table 1:** Clinicopathological features of patients with ILNr and SLNr.

Characteristics	With ILNr(n = 89)	With SLNr(n = 399)	*P* value
Age <60 y, n (%)	42(47.2)	221(55.4)	0.161
Sex, male, n (%)	71(79.8)	288(72.2)	0.142
Neoadjuvant chemotherapy	8(9.0)	52(13.0)	0.294
Tumour site, n (%)			0.498
Upper	42(47.2)	156(39.1)	
Middle	14(15.7)	73(18.3)	
Lower	32(36.0)	160(40.1)	
Whole	1(1.1)	10(2.5)	
Tumour size ≥5 cm, n (%)	54(60.7)	187(46.9)	0.018
Borrmann type III or above, n (%)	66(74.2)	309(77.4)	0.506
Poorly/undifferentiated tumor, n (%)	58(65.2)	278(69.7)	0.407
T stage, n (%)			0.059
T2	0(0.0)	9(2.3)	
T3	14(15.7)	98(24.6)	
T4	75(84.3)	292(73.2)	
N stage, n (%)			< 0.001
N0	10(11.2)	35(8.8)	
N1	37(41.6)	50(12.5)	
N2	29(32.6)	149(37.3)	
N3	13(14.6)	165(41.4)	
TNM stage, n (%)			0.001
IIIA	22(24.7)	106(26.6)	
IIIB	49(55.1)	140(35.1)	
IIIC	18(20.2)	153(38.3)	
Senior surgeon, n (%)	46(51.7)	308(77.2)	< 0.001
CEA >5 ug/L, n (%)	11(12.4)	57(14.3)	0.635
Surgical complication, n ^a^ (%)	19(21.3)	76(19.0)	0.602
Surgical site infection, n (%)	11(12.4)	57(14.3)	0.635
Anastomotic stricture, n (%)	3(3.4)	18(4.5)	0.632
Anastomotic leakage, n (%)	7(7.9)	44(11.0)	0.378
Anastomotic bleeding, n (%)	4(4.5)	15(3.8)	0.746
Abdominal bleeding, n (%)	1(1.1)	5(1.3)	0.920
Pleural effusion, n (%)	3(3.4)	17(4.3)	0.702

^a^n = the number of patients with surgical complications, one patient could simultaneously develop more than one type of complications; ILNr, insufficient lymph nodes removed; SLNr, sufficient lymph nodes removed.

### Analysis of Survival and Its Influencing Factors

**Figure 2:**
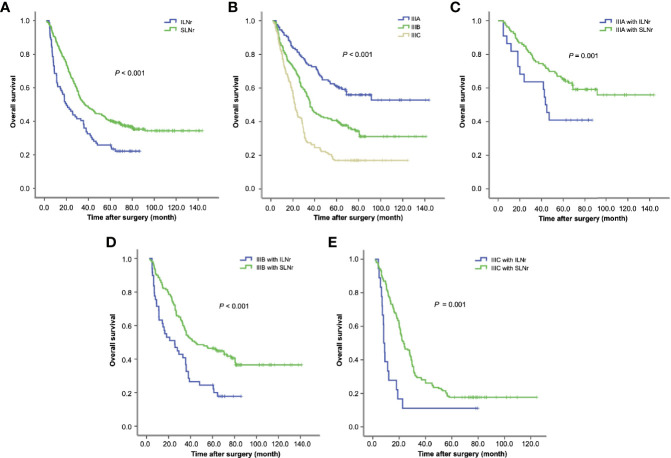
Survival of gastric cancer patients with ILNr and SLNr. **(A)** The OS of stage III patients with SLNr and ILNr. **(B)** The OS of patients with stage IIIA, IIIB, and IIIC gastric cancer; **(C)** The OS of stage IIIA patients with SLNr (n = 106) and ILNr (n = 22); **(D)** The OS of stage IIIB patients with SLNr (n = 140) and ILNr (n = 49); **(E)** The OS of stage IIIC patients with SLNr (n = 153) and ILNr (n = 18). OS, overall survival; SLNr, sufficient lymph nodes removed (≥16 lymph nodes); ILNr, insufficient lymph nodes removed (<16 lymph nodes).

Kaplan-Meier analysis showed that the median survival time of patients with SLNr was significantly longer than that of patients with ILNr (37.7 vs. 21.7 months, *P* < 0.001, **Figure 2A**). Univariate survival analysis revealed that age, tumour size, gross type, histological type, T stage, N stage, TNM stage, status of LNs removed, and the surgeon’s experience were potential factors that influenced OS. Multivariate Cox regression analysis identified larger tumour size, more advanced N, and more advanced TNM stage as the independent impairing factors for OS, while the operations performed by senior surgeons and SLNr (all *P* < 0.05) were the independent protective factors for OS (**Table 2**).

**Table 2:** Uni- and multi-variate analyses of the factors influencing overall survival.

Variables	Univariate Cox regression analysis				Multivariate Cox regression analysis			
	χ^2^ value	OR	95%CI	*P* value	Wald value	HR	95%CI	*P* value
Age >60 y	5.572	1.301	1.046–1.619	0.018				
Male gender	0.033	1.203	0.801–1.307	0.855				
Neoadjuvant chemotherapy	0.033	0.970	0.696–1.350	0.855				
Proximal tumor location	1.486	0.930	0.827–1.045	0.223				
Tumor ≥5 cm	10.597	1.440	1.156–1.793	0.001	6.369	1.415	1.081-1.852	0.012
Borrmann III & IV	7.630	1.475	1.119–1.943	0.006				
Poorly/undifferentiated tumor	3.950	1.279	1.003–1.630	0.047				
Advanced T stage	5.556	1.382	1.056–1.810	0.018				
Advanced N stage	12.038	2.323	1.443-3.739	0.001	4.926	2.147	1.093-4.216	0.026
Advanced TNM stage	61.280	1.812	1.561–2.102	<0.001	44.224	1.929	1.589-2.341	<0.001
SLNr (≥16 LNs)	14.630	0.594	0.455–0.776	<0.001	10.385	0.579	0.415-0.807	0.001
Senior surgeon	8.814	0.699	0.552–0.885	0.003	13.530	0.563	0.414-0.765	<0.001
CEA >5 ug/L	3.373	0.729	0.520–1.023	0.066				
Surgical complication	0.437	1.095	0.837–1.433	0.509				

SLNr, sufficient lymph nodes removed (≥ 16 lymph nodes); OR, odds ratio; CI, confidence interval; HR, hazard ratio.

### Subgroup Analysis of Survival

The OS was markedly reduced with the upstaging from IIIA, IIIB to IIIC (60.0 vs. 37.2 vs. 22.1 months; IIIA vs. IIIB, χ^2^ = 16.8, *P* < 0.001; IIIB vs. IIIC, χ^2^ = 20.9, *P* < 0.001; **Figure 2B**). Having demonstrated that the status of LNs removed impacted the survival of stage III patients, we further determined the impact of the status of LNs removed on substage IIIA, IIIB and IIIC respectively. Stratified analyses for each substage of cases consistently revealed that patients with SLNr exhibited significantly longer OS than those with ILNr (for stage IIIA, 68.2 vs. 43.2 months, χ^2^ = 4.13, *P* = 0.042, **Figure 2C**; for IIIB, 43.7 vs. 24.9 months, χ^2^ = 14.15, *P* < 0.001, **Figure 2D**; and for IIIC, 23.9 vs. 8.3 months, χ^2^ = 11.925, *P* < 0.001, **Figure 2E**).

**Figure 3:**
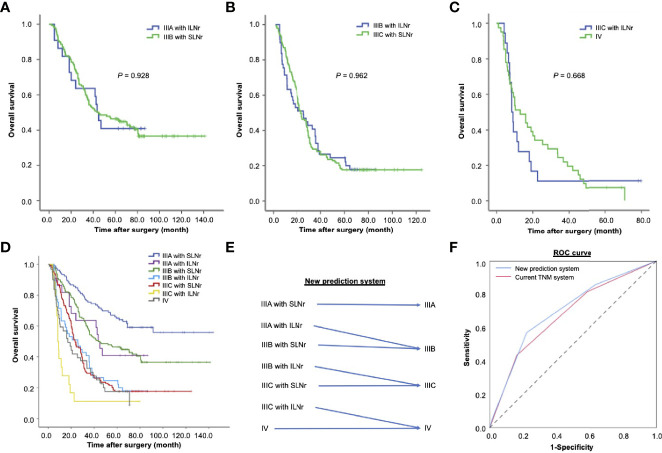
The impact of the status of lymph nodes removed on the prognosis of patients with stage III gastric cancer. **(A)** The OS of stage IIIA patients with ILNr (n = 22) and stage IIIB patients with SLNr (n = 189); **(B)** The OS of stage IIIB patients with ILNr (n = 49) and IIIC patients with SLNr (n = 153); **(C)** The OS of IIIC patients with ILNr (n = 18) and IV patients (n = 41). **(D)** Collective survival curves for different subsets of patients with stage III and stage IV gastric cancer; **(E)** A better prediction system by incorporating the status of lymph nodes removed into TNM staging. **(F)** The receiver operating characteristics curves of the new prediction system and the current TNM system. OS, overall survival; SLNr, sufficient lymph nodes removed (≥ 16 lymph nodes); ILNr, insufficient lymph nodes removed (< 16 lymph nodes).

Notably, the OS was not significantly different between stage IIIA patients with ILNr and stage IIIB patients with SLNr (43.2 vs. 43.7 months, χ^2^ = 0.008, *P* = 0.928, **Figure 3A**). Similarly, no significant difference in OS was found between patients with stage IIIB disease with ILNr and those with stage IIIC with SLNr (24.9 vs. 23.9 months, χ^2^ = 0.002, *P* = 0.962, **Figure 3B**), indicating that upstaging might better reflect the prognosis of patients with ILNr. To examine whether a substage increase in the AJCC system better reflected the prognosis of stage IIIC with ILNr, we reviewed 41 consecutive cases with stage IV gastric cancer, who underwent surgical treatment with curative intent during the same period. Consistently, the OS of stage IIIC patients with ILNr was not significantly different from that of stage IV patients (8.3 vs. 13 months, χ^2^ = 0.184, *P* = 0.668, **Figure 3C**), confirming that a substage increase improved the prognostic prediction for stage III patients with ILNr.

### Evaluation of a New Hypothesized Prediction System

The collective curves of OS for patients with stage IIIA, IIIB, IIIC, and IV tumours are shown in **Figure 3D**. Since the prognosis of patients with ILNr was better reflected by a substage increase in the AJCC classification system, we proposed a new prediction system by integrating the AJCC staging system with the status of LNs removed (**Figure 3E**). In this hypothesize system, one substage was increased in the AJCC classification system, from IIIA to IIIB, from IIIB to IIIC, and from IIIC to IV in patients with ILNr. Compared with the current TNM staging system, we found that the new prediction system had a higher Likelihood ratio χ^2^ (188.6 vs. 184.8), indicating that a higher homogeneity in OS of patients within each substage classified by the new prediction system; the AIC value was lower (4336.4 vs 4340.6), reflecting that the new system had less information lost in prediction of prognosis, while the C-index was higher, supporting a higher proportion of correct predictions in the new system (0.695 vs. 0.679, *P* = 0.002). The ROC curves revealed that the performance of prognostic prediction was better in the new prediction system (AUC = 0.699) compared with the current TNM system (AUC = 0.676) (**Figure 3F**).

### Risk Factors of ILNr

Given that ILNr plays a detrimental role in the OS of stage III gastric cancer, we evaluated on the independent factors that affected the status of LNs removed. Multivariate logistic regression analysis indicated that a large tumour size, more advanced T, and more advanced TNM stage were the independent risk factors for ILNr, while operations performed by senior surgeons was an independent protective factor (**Table 3**).

**Table 3:** Stepwise multivariate logistic regression analysis of risk factors for ILNr.

Variables	Wald test	OR	95%CI	*P* value
Tumor ≥5 cm	4.861	1.812	1.068–3.077	0.027
Advanced T stage	3.732	2.088	0.989–4.405	0.053
Advanced TNM stage	12.548	1.600	1.235–2.075	<0.001
Senior surgeon	10.874	0.408	0.240–0.695	0.010

Variables here were the ones with P < 0.1 in **Table 1**; OR, odds ratio; CI, confidence interval.

## Discussion

Sufficient lymphadenectomy in patients with gastric cancer is difficult. Less than 16 of LNs removed is not sufficient to determine whether the patient reaches N3 stage because 16 is the minimum number required for N3 diagnosis. To increase the accuracy of N staging under this circumstance, some researchers have proposed the N-ratio and have proven its correlation with cancer survival (16–18). However, not satisfying the Bayes’ rule and binomial distribution inevitably make this index to under- or over-estimate the N stage (19). There is still a lack of ideal methods to accurately diagnosed the N stage when LN removal is insufficient. Inaccurate diagnosis of the N stage can hinder the prognostic prediction for patients. This was reflected by the findings of our study, that patients with ILNr had a larger proportion of underestimation for N (*P* < 0.001) and TNM stage (*P* = 0.001). Importantly, for stage III gastric cancer, the patients with ILNr had significantly worse long-term survival than those with SLNr. Subgroup analyses concordantly demonstrated the negative association between ILNr and OS in patients with stage IIIA, IIIB and IIIC diseases, respectively. Moreover, the Cox proportional hazards model highlighted that the status of LNs removed was an independent risk factor that affected the OS of patients. Collectively, our study indicates that the status of LNs removed during curative surgery is an instrumental factor in the prediction of long-term survival of patients with stage III gastric cancer.

Interestingly, we find that there was no significant difference in OS between stage IIIA patients with ILNr and stage IIIB patients with SLNr, stage IIIB patients with ILNr and stage IIIC patients with SLNr, stage IIIC patients with ILNr and stage IV patients, respectively. This underscored the need for a substage increase in the AJCC classification system for patients with ILNr so as to improve the accuracy in predicting prognosis in patients with stage III gastric cancer. To meet the need for better prognostic prediction, we propose a new prediction system for stage III gastric cancer by taking the status of LNs removed into account. In the new system, the stage of patients with SLNr remained unchanged in reference to AJCC cancer staging system; however, if a patient has ILNr, with the number of LNs removed being less than 16, the patient’s tumour stage will be upstaged with one substage increase from IIIA to IIIB, from IIIB to IIIC, and from IIIC to IV. By comparing the performance of different models using the likelihood ratio χ^2^ test, AIC, c-index, and ROC curve, we demonstrated the superiority of the new system in predicting long-term survival over the current AJCC staging system.

Additionally, we found that a larger tumour diameter and more advanced TNM stage were the independent risk factors for ILNr, while surgeries performed by senior surgeons was an independent protective factor. Senior surgeons perform more proficient lymphadenectomy, and this could partially explain the lower proportion of ILNr, which was demonstrated to be detrimental to long-term survival in our study. This notion was supported by other studies which indicate that the experience of surgeons affected the survival of gastric cancer patients (20). Although some studies have argued that surgeons performing ≥ 20 gastrectomies or < 20 gastrecomies did not affect patients’ survival (21), we still tend to believe that senior surgeons will enhance the surgical quality of lymphadenectomy and improve patient prognosis. This difference might be observed when the cut-off number of gastrectomies increases further because 20 is quite a small number compared to the cases of gastrectomies performed over a period of 10 years or more. However, what the exact cut-off value needs further determination to improve survival outcomes. Together, operations performed by senior surgeons should be recommended for the individuals with large size, advanced T, and advance TNM stage of tumours.

This study has some limitations. First, although consecutive patients were included in the analysis, interpretation of the findings should be done cautiously due to the retrospective nature of this study. It is difficult to investigate the effect of ILNr on survival in a prospective study. Ethically, we should remove a sufficient number of LNs for better accurate N staging and we cannot set the ILNr arm for prospective observation. Second, we only analysed the OS of patients but did not observe the relapse-free survival in this study. Third, for stage IB and II gastric cancer with possible LN metastasis, whether ILNr impairs the long-term survival is still unclear. Additionally, despite the inclusion of a small proportion of patients with neoadjuvant therapy in this analysis, we did not observe the impact of neoadjuvant chemotherapy on the long-term survival of patients in our cohort. This might be due to the insufficient sample size analysed. Further, the included population was confined to the postoperative stage III diseases whose response to neoadjuvant chemotherapy might not be as good as expected. In future, we will extend the analysis to patients with other stages of tumours beyond stage III and provide a broader view of the impact of LNs removal on the prognosis of gastric cancer.

In conclusion, ILNr (the number of LNs <16) impairs the long-term outcomes of stage III gastric cancer. One substage increase in the AJCC classification system enhances the accuracy of prediction of prognosis with ILNr. Surgical resection performed by senior surgeons is recommended for patients with large tumours or with more advanced TNM stages.

## Data Availability Statement

The raw data supporting the conclusions of this article will be made available by the authors, without undue reservation.

## Ethics Statement

The studies involving human participants were reviewed and approved by First Affiliated Hospital of Sun Yat-sen University. The patients/participants provided their written informed consent to participate in this study.

## Author Contributions

R-SZ and Y-NL contributed equally to this study, including study concept and design, analysis and interpretation of data, drafting of the manuscript, and critical revision of the manuscript for important intellectual content. W-GD, S-LC, E-TZ, and J-NY participated in the acquisition of data and statistical analysis. J-HC and S-RC supervised the whole study, monitored the standard surgical operations, and edited and reviewed the manuscript. All the authors took part in the surgical treatments for gastric cancer. All authors contributed to the article and approved the submitted version.

## Funding

This study was supported by research grants from the National Natural Science Foundation of China (No.81702878), Guangdong Basic and Applied Basic Research Foundation (2020A1515010214), Project 5010 of The First Affiliated Hospital of Sun Yat-sen University (2018004), and Basic scientific research funding of Sun Yat-sen University (14ykpy58).

## Conflict of Interest

The authors declare that the research was conducted in the absence of any commercial or financial relationships that could be construed as a potential conflict of interest.
